# Vitamin D deficiency and subsequent risk of obstructive sleep apnea: a multi-institutional retrospective study

**DOI:** 10.3389/fnut.2025.1651712

**Published:** 2025-11-12

**Authors:** Kuo-Chuan Hung, Ting-Sian Yu, Yi-Chen Lai, Chih-Wei Hsu, Ming Yew, Chia-Hung Yu, I-Wen Chen

**Affiliations:** 1 Department of Anesthesiology, Chi Mei Medical Center, Tainan, Taiwan; 2 School of Medicine, College of Medicine, National Sun Yat-sen University, Kaohsiung, Taiwan; 3 Department of Anesthesiology, E-Da Hospital, I-Shou University, Kaohsiung, Taiwan; 4 Department of Psychiatry, Kaohsiung Chang Gung Memorial Hospital and Chang Gung University College of Medicine, Kaohsiung, Taiwan; 5 Department of Anesthesiology, Chi Mei Medical Center, Liouying, Tainan, Taiwan

**Keywords:** obstructive sleep apnea, vitamin D deficiency, 25-hydroxyvitamin D, sleep disorders, propensity score matching, dose–response relationship

## Abstract

**Background:**

Obstructive sleep apnea (OSA) is a global health concern associated with cardiovascular and metabolic complications. Vitamin D deficiency (VDD) is common and may contribute to OSA pathophysiology; however, most evidence is cross-sectional. This study investigated whether sustained VDD is associated with an increased risk of developing OSA.

**Methods:**

This retrospective cohort study utilized data from the TriNetX federated research network (2010–2023) to investigate the relationship between VDD and incident OSA in adults aged ≥ 18 years. Patients were classified based on sustained serum 25-hydroxyvitamin D levels, with deficiency defined as ≤20 ng/mL and sufficiency as ≥30 ng/mL. To ensure an accurate exposure status, all included individuals underwent confirmatory vitamin D measurements within 3–12 months. After 1:1 propensity score matching, patients were followed for up to 5 years for new-onset OSA diagnoses, with a three-month washout period after vitamin D assessment to mitigate reverse causality and enhance causal inference.

**Results:**

Analysis of 126,563 matched pairs demonstrated that OSA risk was significantly higher in the VDD group than in the controls (5.7% vs. 4.4%; HR 1.26, 95% CI 1.21–1.30; *p* < 0.001). This association demonstrated temporal consistency across 1-, 3-, and 5-year follow-ups. A clear dose–response relationship emerged, with severe VDD [≤10 ng/mL] conferring greater risk (HR 1.39, 95% CI 1.26–1.53; *p* < 0.001). Subgroup analyses revealed significant effect modification: stronger associations in women versus men (HR 1.32 vs. 1.19; P for interaction: 0.003), younger versus older adults (HR 1.45 vs. 1.15; P for interaction <0.001), and overweight/obese versus normal-weight individuals (HR 1.27 vs. 1.02; P for interaction <0.001).

**Conclusion:**

Sustained VDD was independently associated with OSA risk, with temporal consistency and dose–response relationships supporting potential causality. The effects were most pronounced in women, younger adults, and overweight/obese individuals, suggesting that targeted assessment and intervention strategies may be warranted in these high-risk subgroups.

## 1 Introduction

Obstructive sleep apnea (OSA) is a prevalent and potentially life-threatening sleep disorder affecting an estimated 936 million adults aged 30–69 years worldwide, contributing significantly to global morbidity and healthcare costs (1, 2). Anatomical factors are fundamental to OSA pathogenesis. Dempsey et al. established that variations in upper airway structure and collapsibility play a central role in sleep-disordered breathing, even in non-clinical populations (3). Characterized by repetitive upper airway collapse during sleep, OSA leads to intermittent hypoxemia, sleep fragmentation, and an increased risk of cardiovascular disease, metabolic dysfunction, and neurocognitive impairment (4–8). The economic burden of OSA extends far beyond direct treatment costs, with recent analyses indicating that OSA-related comorbidities and consequences impose healthcare expenditures exceeding €30 billion annually in developed countries, underscoring its substantial impact (9). Recent findings by Gong et al. revealed that body mass index (BMI) serves as a mediator in the link between serum vitamin D levels and sleep deprivation, emphasizing the need to consider metabolic status when assessing the influence of nutritional factors on sleep (10). While traditional risk factors include obesity, male sex, and advancing age (11–14), emerging evidence suggests that nutritional deficiencies may contribute to OSA pathogenesis through mechanisms involving upper airway muscle function, inflammatory pathways, and respiratory control (15–19).

Vitamin D deficiency (VDD), defined as 25-hydroxyvitamin D levels below 20 ng/mL (20), affects approximately 35% of people globally and has been implicated in various respiratory conditions beyond its classical role in bone metabolism (21–24). Mechanistically, vitamin D receptors are widely distributed throughout the respiratory muscles, and VDD may impair upper airway dilator muscle function, increase systemic inflammation, and alter immune responses that could predispose to OSA development (19, 25). While evidence suggests an association between vitamin D status and OSA risk, most studies have been cross-sectional in design, failing to establish temporal relationships and providing limited evidence for causality (26–28). Recent prospective evidence from the UK Biobank demonstrated an inverse association between serum 25-hydroxyvitamin D concentrations and new-onset OSA, particularly in individuals with obesity (29). However, significant knowledge gaps remain, including limited population diversity, uncertain dose–response relationships across different VDD severities, and incomplete understanding of effect modification by demographic and clinical factors beyond obesity. Additionally, while the UK Biobank study provided valuable long-term evidence with a 12-year follow-up (29), it lacked shorter-term outcome assessments that could inform immediate public health intervention strategies.

Given the high prevalence of both VDD and OSA, establishing whether VDD is a modifiable risk factor for OSA development has significant clinical and public health implications. This multi-institutional retrospective study utilized the TriNetX federated health research network to examine the association between sustained VDD and subsequent OSA development in a large, well-characterized patient population using rigorous propensity score matching. We hypothesized that patients with confirmed VDD would demonstrate an increased risk of developing OSA compared to vitamin D-sufficient controls, with potential dose–response relationships and differential effects across patient subgroups.

## 2 Methods

### 2.1 Data sources and ethical statement

This multi-institutional retrospective study used the TriNetX database to identify eligible patients across multiple healthcare networks. TriNetX is a global federated health research network that provides access to de-identified electronic health records from participating healthcare organizations. It has been widely used in previous studies across various medical disciplines, including research on perioperative outcomes, chronic disease management, and health disparities (30–33). The database maintains patient privacy through robust de-identification processes that comply with applicable privacy regulations while preserving data utility for research purposes. The study protocol adhered to the ethical principles outlined in the Declaration of Helsinki for medical research involving human participants. The research protocol was reviewed and approved by the Institutional Review Board of Chi Mei Medical Center (IRB number: 11310-E04), which waived the requirement for informed consent owing to the retrospective nature of the study and the use of de-identified data.

### 2.2 Inclusion criteria

The study period encompassed January 1, 2010, through December 31, 2023, and included patients who were 18 years of age or older at the time of enrollment. Patients were eligible for inclusion if they were identified as having VDD, defined as 25-hydroxyvitamin D [25(OH)D] levels less than 20 ng/mL at their first measurement (VDD group). The index date was defined as the date of the first documented VDD. To ensure that patients had sustained VDD rather than transient low levels, a confirmatory 25(OH)D measurement showing continued deficiency was required within 3 to 12 months following the initial test. Similarly, the control group consisted of patients with vitamin D sufficiency, defined as 25(OH)D levels greater than 30 ng/mL, who also required a second confirmatory measurement within the same 3–12 month timeframe to ensure sustained adequate vitamin D status.

### 2.3 Exclusion criteria

**Figure 1:**
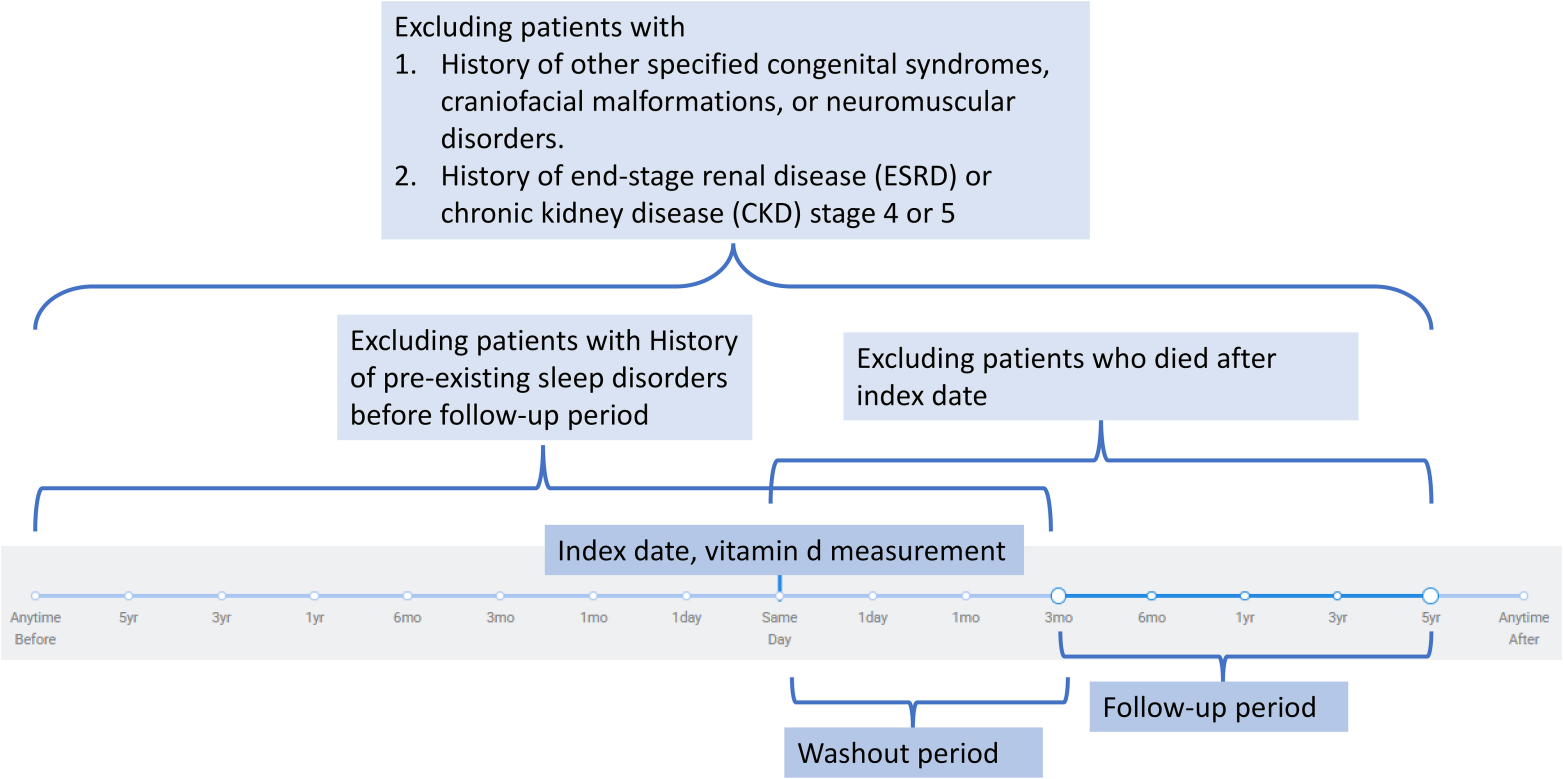
Flowchart illustrating the exclusion criteria and study timeline. Patients were excluded if they had congenital or neuromuscular syndromes, advanced chronic kidney disease (stage 4–5) or end-stage renal disease, pre-existing sleep disorders before follow-up, or death after the index date. The index date was defined as the date of vitamin D measurement. A washout period followed the index date to minimize reverse causality, after which eligible participants were observed during the defined follow-up period.

Patients were excluded if they had any pre-existing sleep disorders at baseline, including insomnia (ICD-10: G47.0), hypersomnia (G47.1), circadian rhythm sleep disorders (G47.2), or sleep apnea (G47.3). Additional exclusion criteria included congenital conditions that could predispose to sleep disorders: other specified congenital malformation syndromes affecting multiple systems (Q87), other congenital malformations of the skull and face bones (Q75), cleft lip and cleft palate (Q35–Q37), and diseases of the myoneural junction and muscle (G70–G73). Patients with advanced kidney disease, including end-stage renal disease (N18.6), chronic kidney disease stage 4 (N18.4), and chronic kidney disease stage 5 (N18.5), were excluded. Other exclusions included acromegaly and pituitary gigantism (E22.0), disorders of the nose and nasal sinuses (J34), and malignant neoplasms of the head, face, and neck (C76.0). Patients who died during the follow-up period between the index date and the study endpoint were also excluded from the analysis. The timeframe for the exclusion criteria is illustrated in Figure 1.

### 2.4 Outcomes and follow-up period

The primary outcome was the development of OSA during the five-year follow-up period after the index date. To minimize bias and reduce misclassification of pre-existing OSA events, a three-month washout period was implemented after the index date, during which any OSA diagnoses were excluded from the analysis. This washout period aimed to better reflect the incident risk attributable to VDD rather than undiagnosed preexisting conditions. The secondary outcomes included the development of other sleep disorders, specifically insomnia (G47.0), hypersomnia (G47.1), and circadian rhythm sleep disorders (G47.2). To assess the short-term effects of VDD, additional analyses were conducted at one-year and three-year follow-up intervals.

### 2.5 Data collection

The baseline demographic, clinical, and medication characteristics of all eligible patients were extracted from the TriNetX database and are summarized in Table 1. Variables included age; sex; race/ethnicity; comorbidities (e.g., cardiovascular, metabolic, renal, pulmonary, hepatic, and oncologic conditions); lifestyle and nutritional factors (e.g., nicotine dependence, alcohol-related disorders, malnutrition, and obesity); and relevant medication use, including antidiabetic agents, sedatives, and vitamin D supplementation.

**Table 1:** Baseline characteristics of patients before and after propensity score matching.

Variables	Before matching			After matching		
	VDD group (*n* = 155,224)	Control group (*n* = 348,470)	SMD^†^	VDD group (*n* = 126,563)	Control group (*n* = 126,563)	SMD^†^
Patient characteristics						
Age at index (years)	46.0 ± 19.1	58.2 ± 17.7	0.660	49.2 ± 18.5	49.4 ± 18.9	0.009
BMI 20–30 kg/m^2^	33,819 (21.8%)	112,701 (32.3%)	0.239	29,267 (23.1%)	28,632 (22.6%)	0.012
BMI 30–40 kg/m^2^	28,407 (18.3%)	55,924 (16.0%)	0.060	22,000 (17.4%)	22,376 (17.7%)	0.008
BMI 40–50 kg/m^2^	10,225 (6.6%)	14,065 (4.0%)	0.114	7,363 (5.8%)	7,614 (6.0%)	0.008
BMI > 50 kg/m^2^	3,116 (2.0%)	3,312 (1.0%)	0.088	2057 (1.6%)	2054 (1.6%)	<0.001
Female	103,580 (66.7%)	249,663 (71.6%)	0.107	85,065 (67.2%)	84,725 (66.9%)	0.006
White	63,912 (41.2%)	242,093 (69.5%)	0.594	62,203 (49.1%)	61,765 (48.8%)	0.007
Factors influencing health status and contact with health services	68,490 (44.1%)	164,167 (47.1%)	0.060	54,251 (42.9%)	53,498 (42.3%)	0.012
Comorbidities						
Essential (primary) hypertension	31,107 (20.0%)	94,104 (27.0%)	0.165	26,033 (20.6%)	26,341 (20.8%)	0.006
Dyslipidemia	25,627 (16.5%)	97,962 (28.1%)	0.281	22,904 (18.1%)	23,202 (18.3%)	0.006
Neoplasms	17,561 (11.3%)	59,089 (17.0%)	0.163	15,341 (12.1%)	15,144 (12.0%)	0.005
Overweight and obesity	21,582 (13.9%)	30,862 (8.9%)	0.159	14,815 (11.7%)	14,983 (11.8%)	0.004
Diabetes mellitus	18,289 (11.8%)	39,859 (11.4%)	0.011	14,441 (11.4%)	14,431 (11.4%)	<0.001
Malaise and fatigue	11,195 (7.2%)	30,065 (8.6%)	0.052	9,301 (7.3%)	9,352 (7.4%)	0.002
Hypothyroidism	9,414 (6.1%)	42,022 (12.1%)	0.210	8,717 (6.9%)	8,740 (6.9%)	0.001
Other anemias	8,780 (5.7%)	17,597 (5.1%)	0.027	6,406 (5.1%)	6,407 (5.1%)	<0.001
Nicotine dependence	8,540 (5.5%)	10,562 (3.0%)	0.122	5,586 (4.4%)	5,637 (4.5%)	0.002
Ischemic heart diseases	5,182 (3.3%)	18,231 (5.2%)	0.094	4,504 (3.6%)	4,513 (3.6%)	<0.001
Diseases of liver	5,960 (3.8%)	10,553 (3.0%)	0.045	4,489 (3.5%)	4,398 (3.5%)	0.004
Chronic kidney disease (CKD)	4,464 (2.9%)	17,837 (5.1%)	0.115	3,946 (3.1%)	3,974 (3.1%)	0.001
Cerebrovascular diseases	3,155 (2.0%)	9,913 (2.8%)	0.053	2,641 (2.1%)	2,610 (2.1%)	0.002
COPD	2,723 (1.8%)	7,489 (2.1%)	0.029	2,289 (1.8%)	2,254 (1.8%)	0.002
COVID-19	2,615 (1.7%)	6,524 (1.9%)	0.014	2069 (1.6%)	2014 (1.6%)	0.003
Heart failure	2,560 (1.6%)	6,028 (1.7%)	0.006	1991 (1.6%)	1940 (1.5%)	0.003
Alcohol related disorders	2,475 (1.6%)	2,761 (0.8%)	0.074	1,533 (1.2%)	1,473 (1.2%)	0.004
Laboratory data						
Hemoglobin>12 mg/dL	75,655 (48.7%)	163,012 (46.8%)	0.039	61,659 (48.7%)	62,021 (49.0%)	0.006
HbA1c ≥ 7%	12,745 (8.2%)	19,201 (5.5%)	0.107	9,448 (7.5%)	9,567 (7.6%)	0.004
Albumin g/dL (≥3.5 g/dL)	68,332 (44.0%)	154,402 (44.3%)	0.006	54,803 (43.3%)	55,698 (44.0%)	0.014
eGFR>60 mL/min/1.73 m^2^	77,800 (50.1%)	162,037 (46.5%)	0.073	62,220 (49.2%)	62,276 (49.2%)	0.001
Medications						
Sedatives/hypnotics	23,323 (15.0%)	60,009 (17.2%)	0.060	19,012 (15.0%)	19,417 (15.3%)	0.009
Vitamin d supplement	11,297 (7.3%)	52,484 (15.1%)	0.249	10,392 (8.2%)	11,041 (8.7%)	0.018
Biguanides	10,653 (6.9%)	21,584 (6.2%)	0.027	8,218 (6.5%)	8,132 (6.4%)	0.003
Insulins and analogues	10,327 (6.7%)	16,261 (4.7%)	0.086	7,412 (5.9%)	7,437 (5.9%)	0.001
Sulfonylureas	3,978 (2.6%)	7,551 (2.2%)	0.026	3,077 (2.4%)	3,055 (2.4%)	0.001
GLP-1 analogues	2,190 (1.4%)	5,813 (1.7%)	0.021	1820 (1.4%)	1795 (1.4%)	0.002
DPP-4 inhibitors	2026 (1.3%)	4,918 (1.4%)	0.009	1,699 (1.3%)	1,667 (1.3%)	0.002
SGLT2 inhibitors	1,325 (0.9%)	4,073 (1.2%)	0.032	1,158 (0.9%)	1,169 (0.9%)	0.001

VDD, vitamin D deficiency; BMI, body mass index; SMD, standardized mean differences; ^†^ SMD values <0.1 indicate adequate balance between groups; eGFR, estimated Glomerular Filtration Rate; COPD, chronic obstructive pulmonary disease; GLP-1, Glucagon-like peptide-1; DPP-4, Dipeptidyl peptidase 4; SGLT2, Sodium-glucose co-transporter 2.

### 2.6 Subgroup analyses

Pre-specified subgroup analyses were conducted to examine the consistency of the primary findings across different patient populations. Subgroups were defined by age (18–50 years versus >50 years), sex (male versus female), presence of diabetes mellitus (yes versus no), presence of hypertension (yes versus no), presence of chronic kidney disease (yes versus no), and BMI categories (BMI < 25 kg/m^2^ versus BMI ≥ 25 kg/m^2^). These analyses aimed to identify potential effect modifiers and assess the generalizability of the findings across diverse patient populations.

### 2.7 Additional analyses

To further characterize the relationship between vitamin D status and sleep disorders, we evaluated the impact of severe VDD, defined as 25(OH)D levels less than 10 ng/mL, on OSA incidence rates. The rationale for this analysis was to determine whether profound VDD conferred an additional risk beyond moderate deficiency.

### 2.8 Statistical analysis

Continuous variables are presented as mean ± standard deviation, while categorical variables are expressed as frequencies and percentages. To address potential selection bias and ensure balanced comparison groups, one-to-one propensity score matching was performed using a caliper width of 0.1 standard deviations of the logit of the propensity score. The propensity score model included all baseline demographic characteristics, comorbidities, and medications as covariates. Matching was conducted without replacement using a greedy nearest-neighbor algorithm to optimize the balance between groups. Baseline characteristics between the VDD and control groups were compared using standardized mean differences (SMD), with SMD values less than 0.1 indicating adequate balance between matched groups.

Time-to-event analyses were conducted using Kaplan–Meier survival curves, and Cox proportional hazards regression models were employed to estimate hazard ratios (HR) and 95% confidence intervals (CI) for the development of OSA and other sleep disorders. The proportional hazard assumption was assessed using Schoenfeld residuals to ensure model validity. Multivariate Cox proportional hazards models were constructed to identify independent risk factors for new-onset OSA, adjusting for relevant baseline characteristics. To account for multiple comparisons across the four primary outcomes, Bonferroni correction was applied, and statistical significance was set at *p* < 0.0125 (0.05/4 outcomes). All statistical analyses were performed using the built-in analytical tools of the TriNetX platform, ensuring the consistency and reproducibility of results across the federated network.

## 3 Results

### 3.1 Patient selection and baseline characteristics

**Figure 2:**
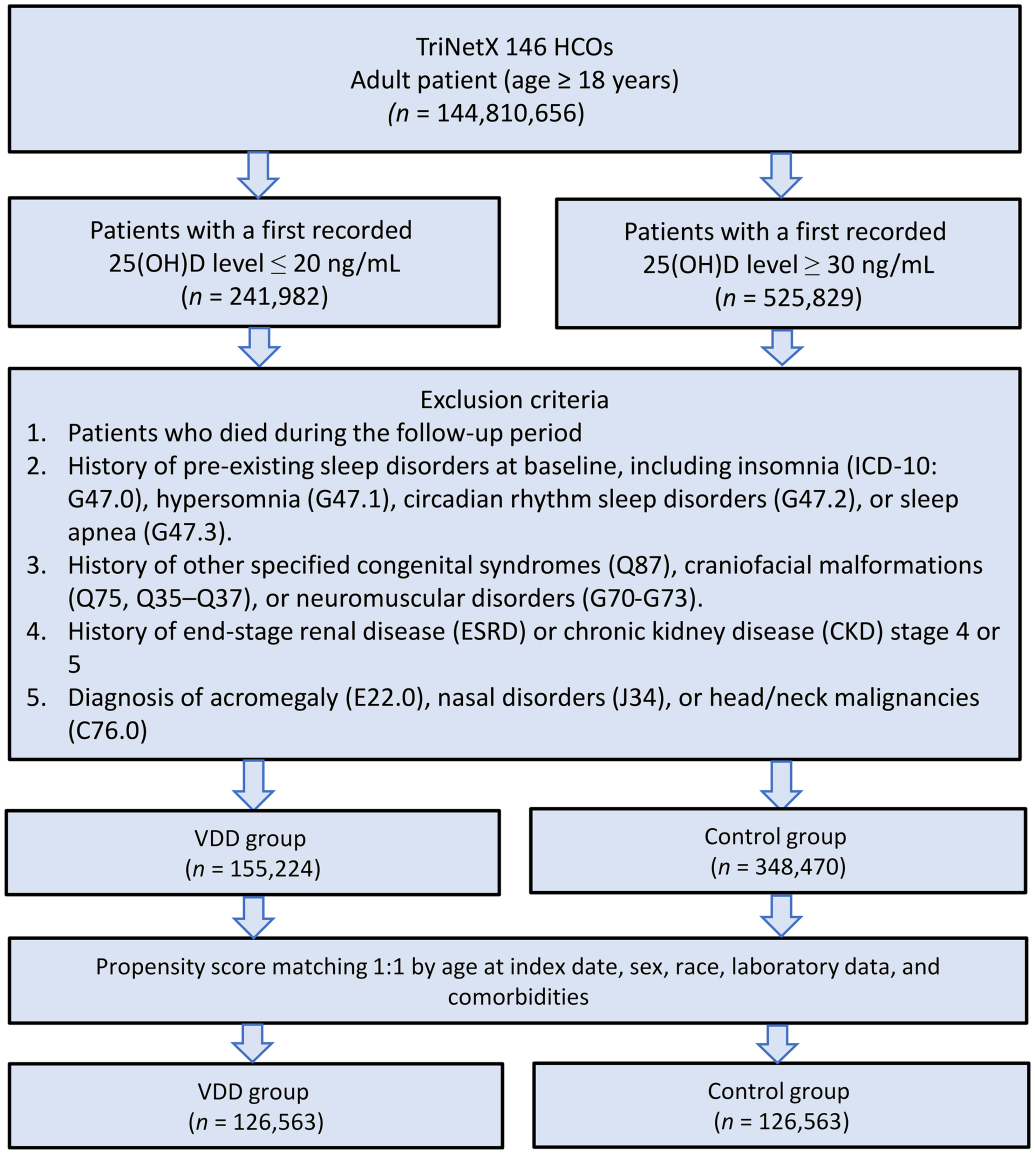
Patient selection flowchart from the TriNetX database. The flowchart illustrates the systematic exclusion process applied to identify eligible patients with vitamin D deficiency (VDD) and vitamin D sufficiency (control group). HCOs, Healthcare Organizations.

**Figure 3:**
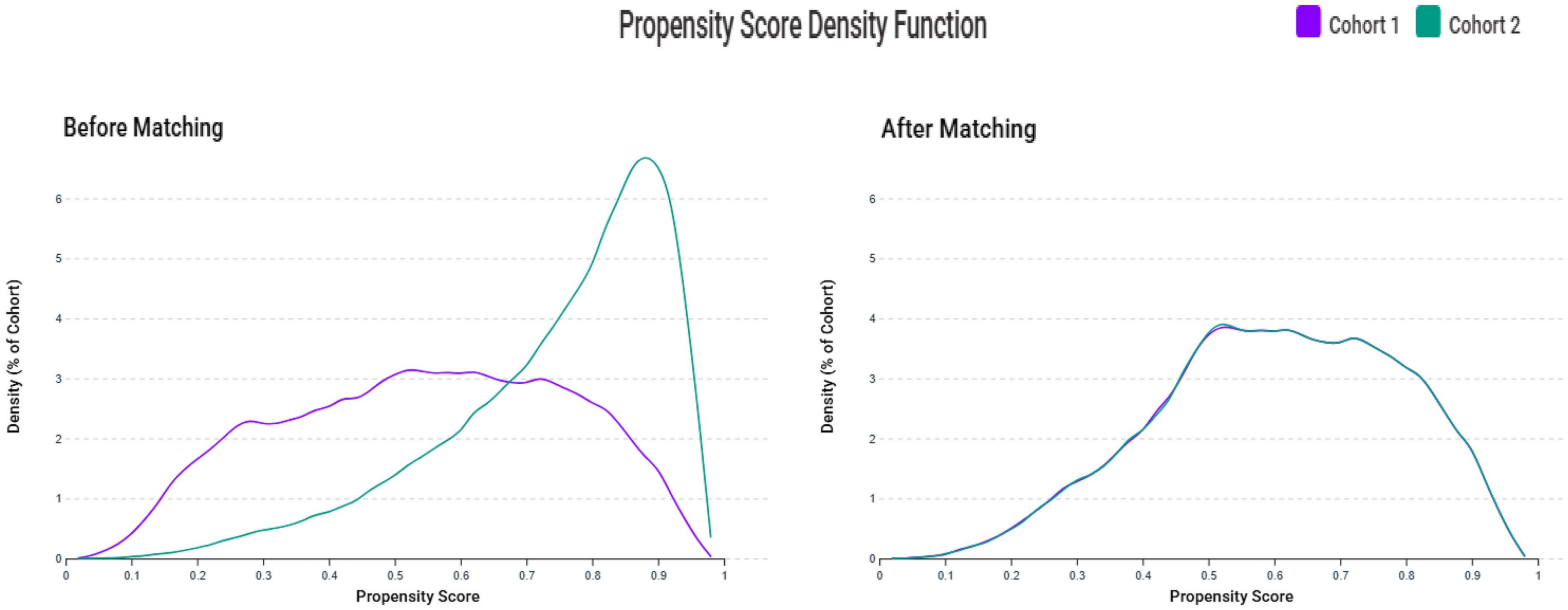
Propensity score density distributions before and after matching. The left panel shows propensity score distributions before matching, demonstrating substantial overlap but different distribution patterns between the vitamin D deficiency group (Cohort 1) and control group (Cohort 2). The right panel shows propensity score distributions after 1:1 matching with a caliper width of 0.1 standard deviations, demonstrating excellent overlap and balance between groups.

The patient selection process is illustrated in Figure 2. After applying exclusion criteria (e.g., pre-existing sleep disorders, advanced kidney disease, congenital abnormalities, and follow-up mortality), we identified an initial cohort of 155,224 patients with documented VDD and 348,470 patients with sufficient vitamin D levels. Propensity score matching was performed using a caliper width of 0.1 standard deviations without replacement. The effectiveness of the matching procedure is illustrated in Figure 3, which demonstrates a substantial overlap between the density functions of the matched groups. After applying 1:1 propensity score matching, each well-balanced cohort comprised 126,563 individuals.

Table 1 presents the detailed baseline characteristics before and after matching. Before matching, the VDD group tended to be younger (46.0 ± 19.1 vs. 58.2 ± 17.7 years) and showed a higher prevalence of overweight/obesity and nicotine dependence, while the control group had higher rates of cardiovascular and metabolic comorbidities (e.g., hypertension and lipid disorders). After matching, the two groups were nearly identical in age (49.2 ± 18.5 vs. 49.4 ± 18.9 years), sex distribution (67.2% vs. 66.9% female), and racial composition (49.1% vs. 48.8% White). Balance was also achieved across a wide range of clinical conditions including diabetes (11.4% in both groups), hypertension (20.6% vs. 20.8%), chronic kidney disease (3.1% in both groups), and chronic obstructive pulmonary disease (1.8% in both groups). In addition, medication use (e.g., sedatives/hypnotics) was well balanced, reducing the risk of confounding due to pharmacological factors.

### 3.2 Outcomes

#### 3.2.1 Risk of outcomes at 5-year follow-up

The primary analysis demonstrated a significant association between VDD and sleep disorders (Table 2). OSA showed the strongest association, with a 5.7% incidence in the VDD group versus 4.4% in controls, corresponding to a 26% increased risk (HR 1.26, 95% CI 1.21–1.30; *p* < 0.001). The secondary outcomes further supported the link between VDD and sleep disruption. Insomnia incidence rose from 4.3 to 5.2% (HR 1.19, 95% CI 1.15–1.23; *p* < 0.001), and hypersomnia from 1.18 to 1.41% (HR 1.15, 95% CI 1.07–1.23; *p* < 0.001). In contrast, circadian rhythm disorders showed no association with vitamin D status (0.25% vs. 0.24%; HR 0.995, 95% CI 0.85–1.16; *p* = 0.952).

**Table 2:** Association between vitamin D deficiency and 5-year outcomes.

Outcomes	VDD group (*n* = 126,563)	Control group (*n* = 126,563)	HR (95% CI)	*P*-value^*^
	Events (%)	Events (%)		
OSA	7,173 (5.7%)	5,522 (4.4%)	1.26 (1.21–1.30)	<0.001
Insomnia	6,629 (5.2%)	5,383 (4.3%)	1.19 (1.15–1.23)	<0.001
Hypersomnia	1,783 (1.41%)	1,489 (1.18%)	1.15 (1.07–1.23)	<0.001
Circadian rhythm sleep disorders	322 (0.25%)	309 (0.24%)	0.995 (0.85–1.16)	0.952

VDD, vitamin D deficiency; OSA, obstructive sleep apnea; HR, hazard ratio; CI, confidence interval; ^*^ *P* < 0.0125 was considered significance following Bonferroni correction.

#### 3.2.2 Risk of outcomes at 1-year and 3-year follow-up

Temporal analyses revealed consistent associations across the follow-up intervals (Table 3). The risk of OSA was evident early (HR 1.25, 95% CI 1.17–1.33; *p* < 0.001 at 1 year) and remained stable at 3 years (HR 1.28, 95% CI 1.23–1.33; *p* < 0.001), reinforcing the likelihood of a causal relationship rather than a chance finding. Insomnia showed similar persistence, with significant associations at 1 year (HR 1.22, 95% CI 1.14–1.30; *p* < 0.001) and 3 years (HR 1.19, 95% CI 1.14–1.24; *p* < 0.001). In contrast, the link between VDD and hypersomnia attenuated over time, declining from a strong association at 1 year (HR 1.25, 95% CI 1.10–1.43; *p* = 0.001) to a weaker but still significant effect at 3 years (HR 1.10, 95% CI 1.02–1.19; *p* = 0.020). Consistently, circadian rhythm sleep disorders showed no significant association with vitamin D status at either the 1-year or 3-year follow-up.

**Table 3:** Analyses of the association between vitamin D deficiency (VDD) and outcomes at 1- and 3-year follow-up.

Outcomes	1-year (*n* = 127,664)		3-year (*n* = 126,768)	
	HR (95% CI)	*P*-values^*^	HR (95% CI)	*P*-values^*^
OSA	1.25 (1.17–1.33)	<0.001	1.28 (1.23–1.33)	<0.001
Insomnia	1.22 (1.14–1.30)	<0.001	1.19 (1.14–1.24)	<0.001
Hypersomnia	1.25 (1.10–1.43)	0.001	1.10 (1.02–1.19)	0.020
Circadian rhythm sleep disorders	088 (0.64–1.22)	0.457	1.09 (0.90–1.32)	0.375

VDD, vitamin D deficiency; OSA, obstructive sleep apnea; HR, hazard ratio; CI, confidence interval; ^*^ *P* < 0.0125 was considered significance following Bonferroni correction.

### 3.3 Association between severe vitamin D deficiency and 5-year outcomes

Analysis of severe VDD demonstrated a clear dose–response relationship, supporting biological plausibility (Table 4). Among 15,180 matched pairs, individuals with severe deficiency (25(OH)D < 10 ng/mL) exhibited a significantly higher risk of OSA than those with moderate deficiency (HR 1.39, 95% CI 1.26–1.53; *p* < 0.001). This 39% increase in hazard notably exceeded the 26% risk associated with moderate deficiency, indicating a graded risk pattern. Similarly, severe deficiency was associated with increased insomnia risk (HR 1.36, 95% CI 1.23–1.50; *p* < 0.001). In contrast, no significant associations were observed for hypersomnia or circadian rhythm disorders, suggesting that these conditions may involve distinct pathophysiological pathways.

**Table 4:** Association between severe vitamin D deficiency and 5-year outcomes.

Outcomes	Severe VDD group (*n* = 15,180)	Control group (*n* = 15,180)	HR (95% CI)	*P*-value^*^
	Events (%)	Events (%)		
OSA	975 (6.4%)	693 (4.6%)	1.39 (1.26–1.53)	< 0.001
Insomnia	908 (6.0%)	659 (4.3%)	1.36 (1.23–1.50)	< 0.001
Hypersomnia	200 (1.3%)	180 (1.2%)	1.08 (0.89–1.33)	0.431
Circadian rhythm sleep disorders	41 (0.3%)	30 (0.2%)	1.32 (0.82–2.11)	0.251

VDD, vitamin D deficiency; OSA, obstructive sleep apnea; HR, hazard ratio; CI, confidence interval; ^*^ *P* < 0.0125 was considered significance following Bonferroni correction.

### 3.4 Subgroup analyses

The effect modification analysis revealed clinically meaningful variations in the association between VDD and OSA risk (Table 5). Notably, sex differences were pronounced, as women exhibited a 32% increased risk of OSA (HR 1.32, 95% CI 1.27–1.38) compared to a 19% increase in men (HR 1.19, 95% CI 1.12–1.25; p for interaction = 0.003), suggesting potential interactions between vitamin D status and sex-specific factors in OSA pathogenesis. Age-stratified analysis demonstrated an inverse relationship with vitamin D sensitivity. Younger adults (18–50 years) experienced a 45% elevated risk (HR 1.45, 95% CI 1.36–1.55), whereas older individuals showed only a modest 15% increase (HR 1.15, 95% CI 1.11–1.20; p for interaction < 0.001), indicating possible age-related variations in vitamin D responsiveness or the influence of competing risk factors. BMI has emerged as a strong effect modifier. No significant association was observed among normal-weight individuals (HR 1.02, 95% CI 0.94–1.13; *p* = 0.562), whereas overweight and obese patients demonstrated a 27% increased risk (HR 1.27, 95% CI 1.22–1.31; *p* < 0.001; p for interaction < 0.001), suggesting that VDD may amplify OSA risk primarily in the context of obesity-related mechanisms.

**Table 5:** Subgroup analyses of association between vitamin D deficiency and risk of obstructive sleep apnea (OSA) at 5-year follow-up.

Subgroup analysis	Number of each group	HR (% CI)	*P*-value^*^	P for interaction^*^
Sex				
Male	42,358	1.19 (1.12–1.25)	<0.001	Reference
female	92,720	1.32 (1.27–1.38)	<0.001	0.003
Age				
18–50 years	50,224	1.45 (1.36–1.55)	<0.001	Reference
>50 years	88,150	1.15 (1.11–1.20)	<0.001	<0.001
Body mass index				
<25 kg/m^2^	33,181	1.02 (0.94–1.13)	0.562	Reference
≥25 kg/m^2^	105,198	1.27 (1.22–1.31)	<0.001	<0.001
Chronic kidney disease				
Yes	12,517	1.11 (1.02–1.21)	0.013	Reference
No	127,318	1.29 (1.25–1.34)	<0.001	0.001
Diabetes Mellitus				
Yes	30,013	1.16 (1.11–1.23)	<0.001	Reference
No	108,574	1.22 (1.17–1.27)	<0.001	0.132
Hypertension				
Yes	52,491	1.19 (1.14–1.24)	<0.001	Reference
No	85,674	1.24 (1.17–1.32)	<0.001	0.277

HR, hazard ratio; CI, confidence interval; ^*^ *P* < 0.05 was considered significance.

### 3.5 Risk factors for OSA

Multivariable modeling confirmed VDD as an independent predictor of OSA (Table 6). Even after adjusting for key confounders, VDD remained significantly associated with increased risk (HR 1.23, 95% CI 1.20–1.27; *p* < 0.001), supporting its potential pathophysiological role beyond residual confounding. Among covariates, obesity demonstrated the strongest association, more than doubling the risk of OSA (HR 2.43, 95% CI 2.36–2.51; *p* < 0.001). Respiratory comorbidities also contributed significantly, with COPD increasing the risk by 55% (HR 1.55, 95% CI 1.45–1.67; *p* < 0.001) and heart failure by 48% (HR 1.48, 95% CI 1.38–1.59; *p* < 0.001). COVID-19 was identified as an emerging predictor (HR 1.40, 95% CI 1.28–1.53; *p* < 0.001), possibly reflecting persistent post-infectious respiratory effects. Male sex was also associated with a higher risk (HR 1.39, 95% CI 1.36–1.43; *p* < 0.001), consistent with known epidemiological trends.

**Table 6:** Risk factors for new-onset obstructive sleep apnea.

Variables	HR (95% CI)^†^	*P*-value
VDD vs. control group	1.23 (1.20, 1.27)	<0.001
Male	1.39 (1.36, 1.43)	<0.001
Age at index	1.00 (1.00, 1.00)	<0.001
COVID-19	1.40 (1.28, 1.53)	<0.001
Overweight and obesity	2.43 (2.36, 2.51)	<0.001
Diabetes mellitus	1.26 (1.22, 1.31)	<0.001
Nicotine dependence	1.08 (1.02, 1.13)	0.014
Chronic kidney disease (CKD)	0.96 (0.91, 1.02)	0.191
Chronic obstructive pulmonary disease	1.55 (1.45, 1.67)	<0.001
Diseases of liver	1.08 (1.02, 1.15)	0.014
Heart failure	1.48 (1.38, 1.59)	<0.001

HR, hazard ratio; CI, confidence interval; ^†^ Cox proportional hazards model.

## 4 Discussion

This large-scale multi-institutional retrospective study demonstrates that patients with sustained VDD faced an increased hazard for OSA development over 5 years. This association demonstrated temporal consistency, a dose–response relationship, and variation across clinically relevant subgroups, supporting a potential causal link. In subgroup analyses, the vitamin D-OSA association was most pronounced in women, younger adults, and individuals with elevated BMI but showed no significant effect in normal-weight individuals. Beyond OSA, VDD is associated with an increased risk of insomnia and hypersomnia, but not circadian rhythm disorders, suggesting specificity in sleep-related pathophysiological mechanisms.

The relationship between vitamin D status and OSA has garnered increasing attention, with accumulating evidence suggesting both cross-sectional associations and potential mechanistic links (26–28). Previous systematic reviews and meta-analyses have consistently demonstrated lower serum 25-hydroxyvitamin D levels in patients with OSA than in controls (26–28). However, most previous studies have been limited by their cross-sectional designs, small sample sizes, and inadequate control for confounding variables, making it difficult to establish temporal relationships or infer causality. Mechanistically, vitamin D support the potential role of vitamin D in OSA pathogenesis is supported by several biological pathways (19, 25). Vitamin D receptors are widely distributed throughout the respiratory muscles, including the upper airway dilator muscles, and deficiency may impair muscle function and reduce upper airway stability during sleep (19, 25). Additionally, vitamin D plays a crucial role in immune regulation and inflammatory pathways, with deficiency associated with increased systemic inflammation that could contribute to upper airway edema and collapsibility (19, 25). The landmark UK Biobank study provided the first large-scale prospective evidence, demonstrating an inverse relationship between serum 25-hydroxyvitamin D concentrations and new-onset OSA over 12 years of follow-up, particularly in individuals with obesity (29).

Our findings demonstrate remarkable temporal consistency in the vitamin D-OSA association across multiple follow-up intervals, suggesting that VDD confers an early and sustained increased risk rather than delayed effects. This consistent temporal pattern supports a potential causal relationship, as random associations are likely to vary across different follow-up periods. The early manifestation of risk within the first year has important clinical implications, suggesting that vitamin D status assessment and potential supplementation may be relevant for short-term OSA prevention strategies. Perhaps the most compelling evidence for a causal relationship comes from our demonstration of a clear dose–response effect. Patients with severe VDD faced a substantially higher OSA risk than those with moderate deficiency, indicating that the relationship is not simply threshold-dependent but exhibits true biological gradation. Such dose–response relationships are considered one of the strongest criteria for establishing causality in observational studies. Our findings align with and substantially extend the UK Biobank results (29), providing novel contributions, including demonstration across diverse healthcare networks, implementation of a three-month washout period to reduce misclassification bias, and requirement for confirmatory vitamin D measurements ensuring sustained deficiency rather than transient low levels.

Our demonstration of significant effect modification across demographic and clinical subgroups represents a novel and potentially paradigm-shifting finding with implications for both mechanistic understanding and clinical practice. The significantly stronger vitamin D-OSA associations in women than in men suggest that vitamin D may interact with hormonal or physiological factors that vary between sexes. Several mechanisms could explain this observation, including higher vitamin D-binding protein levels in women affecting bioavailable vitamin D concentrations, estrogen influences on both vitamin D metabolism and upper airway muscle function, and sex-specific anatomical and physiological determinants of OSA that may be differentially sensitive to vitamin D status (34–36). The striking inverse relationship between age and vitamin D sensitivity represents another novel finding, with younger adults demonstrating a dramatically increased OSA risk compared to older patients. This pattern suggests that VDD may be particularly detrimental during periods of active tissue development and maintenance, with younger individuals potentially having greater vitamin D receptor density and responsiveness. Perhaps the most clinically significant subgroup finding is the complete absence of a vitamin D-OSA association in normal-weight individuals, in contrast with the substantial risk in overweight/obese patients. This interaction suggests that VDD may specifically potentiate obesity-related OSA mechanisms rather than acting as an independent risk factor, with implications including the role of vitamin D in adipose tissue inflammation, metabolic dysfunction, and insulin resistance.

Our multivariable analysis identified VDD as an independent predictor of OSA and clarified its relative contribution alongside established risk factors. Obesity has emerged as the dominant risk factor with overwhelming impact, confirming its central role in OSA pathogenesis and reinforcing that weight management remains the most critical intervention for OSA prevention and treatment. Respiratory comorbidities demonstrate a substantial OSA risk, likely reflecting shared pathophysiological mechanisms, including chronic inflammation and altered respiratory mechanics that predispose to upper airway instability during sleep (37, 38). The emergence of COVID-19 as a significant predictor of OSA in our study aligns with recent findings by Lin et al. (39), who demonstrated that individuals with a history of SARS-CoV-2 infection had a substantially elevated risk of developing OSA during a 12-month follow-up period (HR: 1.57). This contemporary association may reflect the long-term respiratory sequelae of COVID-19, including persistent inflammation, upper airway dysfunction, and post-infectious respiratory compromise. Male sex conferred an increased risk, consistent with established epidemiological patterns reflecting anatomical, hormonal, and behavioral differences (14). Despite adjustment for these multiple confounders, VDD retained a significant association, indicating genuine pathophysiological relevance rather than confounding by other risk factors.

Our analysis extends beyond OSA to explore the broader impact of vitamin D on sleep health, revealing disorder-specific associations that offer mechanistic insights. Insomnia showed a consistent association with VDD across all follow-up periods, with stronger links observed in cases of severe deficiency, suggesting a dose–response relationship comparable to that seen with OSA. The vitamin D-insomnia association aligns with growing evidence for the role of vitamin D in sleep regulation through multiple pathways, including vitamin D receptors in brain regions involved in sleep–wake control and vitamin D’s anti-inflammatory effects that may influence sleep quality through modulation of cytokine pathways affecting sleep architecture (40–42). Hypersomnia showed weaker and less consistent associations, with significant effects that diminished over longer follow-up periods, suggesting that VDD may contribute to the early manifestations of excessive daytime sleepiness through mechanisms involving sleep quality and restorative sleep processes. Notably, circadian rhythm sleep disorders showed no significant association with vitamin D status, suggesting that while vitamin D influences sleep quality and respiratory control during sleep, it may not substantially affect the timing and entrainment of circadian rhythms in clinical populations.

Recent critical reviews have highlighted a complex bidirectional relationship between the vitamin D status and OSA development. Schiza et al. (43) emphasized that while there is growing evidence supporting the role of vitamin D in sleep maintenance and regulation, the fundamental question of causality remains unresolved: whether VDD increases OSA risk or OSA itself predisposes to VDD. This uncertainty underscores the importance of our study design with temporal sequencing and washout periods to strengthen causal inference. The meta-analysis by Serafin et al. (44), which excluded patients with comorbidities, reinforces our findings by showing that the vitamin D–OSA association is independent of systemic confounders. Their focus on otherwise healthy populations supports our observation that VDD may be an independent risk factor rather than merely a consequence of OSA-associated comorbidities. Our study extends these findings by demonstrating that sustained VDD precedes OSA development in a large, well-matched cohort, with consistent effects across multiple follow-up intervals.

Several limitations of our study should be acknowledged when interpreting our findings. First, as this was a retrospective observational study using electronic health records, we could not establish definitive causality despite the temporal sequence and dose–response relationships observed. Unmeasured confounding variables, including dietary patterns, physical activity levels, and socioeconomic factors, may influence both vitamin D status and OSA risk. Second, our reliance on ICD-10 diagnostic codes for outcome ascertainment may introduce misclassification bias because OSA diagnosis in clinical practice varies in accuracy and may be influenced by healthcare access, physician awareness, and patient symptom presentation. Third, vitamin D supplementation, although captured as a baseline characteristic, was not incorporated into stratified analyses or adjusted as a time-varying covariate owing to the limitations of the TriNetX dataset, which lacks reliable information on supplement dosage, duration, adherence, and initiation timing. We opted not to exclude patients who had received supplements, as doing so could introduce selection bias and limit generalizability. However, we recognize that the inability to fully account for supplementation introduces residual confounding, and future studies with more granular data on supplementation patterns are warranted. Fourth, although the TriNetX database provides substantial statistical power and diverse populations, it may not be fully representative of all demographic groups or healthcare systems, potentially limiting its generalizability to resource-limited settings. Finally, our study period encompassed the COVID-19 pandemic, which may have affected both healthcare utilization patterns and sleep disorder diagnosis rates.

## 5 Conclusion

This study demonstrated an independent association between VDD and an increased risk of developing OSA, supported by temporal consistency, dose–response relationships, and biologically plausible effect modification. Our novel finding that vitamin D effects are most pronounced in women, younger adults, and overweight/obese individuals has important implications for personalized risk assessments and targeted intervention strategies. From a clinical perspective, our findings suggest that vitamin D status assessment may be valuable as part of a comprehensive OSA risk evaluation, particularly in women, younger adults, and individuals with an elevated BMI. Future research should focus on randomized controlled trials to definitively establish whether vitamin D supplementation can prevent OSA development, particularly in the high-risk subgroups identified in our analysis.

## Data Availability

The raw data supporting the conclusions of this article will be made available by the authors, without undue reservation.
